# Editorial: Post-traumatic growth

**DOI:** 10.3389/fpsyg.2023.1227892

**Published:** 2023-07-26

**Authors:** Emre Senol-Durak, Marialaura Di Tella, Annunziata Romeo

**Affiliations:** ^1^Department of Psychology, Bolu Abant Izzet Baysal University, Bolu, Türkiye; ^2^Department of Psychology, University of Turin, Turin, Italy

**Keywords:** post-traumatic growth, stress-related growth, loss of loved one, chronic illnesses, post-traumatic depreciation, resilience, natural disasters, violence

Studies on traumatic events have mainly focused on their unfavorable effects, like post-traumatic stress disorder. On the other hand, traumatic encounters that shatter people's presumptions can result in positive experiences. Positive experiences following traumatic events are what Tedeschi and Calhoun (1996, 2004) refer to as “Post-Traumatic Growth” (PTG). Other researchers prefer to refer to the positive changes caused by long-term life experiences as “stress-related growth,” this term is preferred for chronic life events (Park, 2010). PTG includes priorities adjustments, life's purpose, interpersonal connections, inner fortitude, and spirituality. It is important to note that PTG does not negate trauma's negative effects but acknowledges that growth can occur alongside pain and suffering. Following a significant but unpleasant life experience, it is a positive metamorphosis. Beyond simple personality shifts, post-traumatic growth reflects true change, which we may continually learn and benefit from Tedeschi and Blevins (2022). Studies on post-traumatic growth after major life events have shown that the factors contributing to PTG vary depending on the trauma and that there are important therapeutic ramifications for overcoming a difficult experience. In particular, it is not the trauma *per se* that is the catalyst for change but the abrupt disintegration of one's fundamental assumptions and the cognitive process implicated in reestablishing functional assumptions. Studies that investigate the disruption of core beliefs, rumination, coping, and depressive symptoms in relation to PTG are numerous (Senol-Durak and Ayvasik, 2010; Zhou et al., 2015; Senol-Durak and Durak, 2018; Durak and Durak, 2019; Romeo et al., 2020, 2022; David et al., 2022). Also, it is mentioned that individuals, families, and larger social groups may all experience the effects of PTG (Berger, 2015).

Furthermore, cultural influences have a significant impact on PTG. Recently, the “Post-traumatic growth inventory,” a widely used assessment, was altered to take into account diverse cultures and religious affiliations. Furthermore, after PTG, a new notion known as “post-traumatic depreciation” was introduced, which has received little attention in the literature. In this collection of Research Topics, we encourage researchers to contribute to post-traumatic growth and post-traumatic depreciation studies in order to understand better the presence of different types of positive psychological transformations (e.g., natural disasters, accidents, violence, the death of a loved one, chronic illness) in people from other countries. It is important to note that PTG does not negate the pain and suffering experienced during the traumatic event but acknowledges the potential for growth and positive change resulting from it.

Over the past 20 years, PTG has been investigated among individuals suffering from various traumatic events. The term has served different perspectives for professionals working with trauma survivors (Tedeschi and Calhoun, 2004), and recovery from trauma can lead to positive functioning that has been estimated in clinical settings. Besides, contributing factors affecting PTG have been mentioned in those studies, with the theoretical model explaining the rationale behind PTG (Tedeschi and Calhoun, 1996; Senol-Durak and Ayvasik, 2010; Hallam and Morris, 2014). Moreover, the literature discusses trauma survivors' cultural aspects (Calhoun et al., 2010; Tedeschi et al., 2017). Primarily, English-written self-measures assessing PTG, such as the Post-traumatic Growth Inventory, have been investigated in other countries to see cultural parameters in the phenomenon (Tedeschi et al., 2017). Also, a shorter form of PTGI has psychometrically significant results (Platte et al., 2023). Studies have also examined the connection between PTG and other psychological concepts like resilience and coping mechanisms (Tedeschi et al., 2017; Taku et al., 2021). Understanding these relationships can help people learn how to grow from traumatic experiences and cope with them in the best way possible.

We are interested in well-defined research that addresses the current PTG literature. Priority was given during the call for papers to research that (1) investigates post-traumatic growth and its sub-dimensions, such as changes in life priorities, the meaning of life, social relationships, personal strength, and spirituality; (2) tests the theoretical models identified for PTG; (3) focuses on factors contributing to post-traumatic growth based on trauma type; (4) contributes to PTG studies with qualitative, quantitative, and mixed methods; (5) discovers PTG through cultural and cross-cultural studies; (6) applies PTG in clinical settings, and (7) explores post-traumatic depreciation following a profoundly important event. Scholars are specifically asked to submit papers on the following PTG-related topics: (1) PTG with a cultural or cross-cultural focus; (2) PTG and PTD comparisons based on the types, nature, and meanings of specific traumatic events; (3) PTG-related hypotheses and theories, and (4) PTG in clinical/therapeutic settings.

Eleven papers have been published on the topic of post-traumatic growth; nine of them are “original research,” while the other two are “brief research reports”. Findings suggest that post-traumatic growth can take place in a variety of life areas, including interpersonal relationships, spirituality, and self-perception. It is a complicated and multifaceted phenomenon. The studies also emphasize the value of meaning-making, resilience, hope, optimism, post-traumatic stress, cognitive processing, and social support in promoting post-traumatic growth.

The first article, authored by Jaafar et al. and titled “*Post-traumatic growth, positive psychology, perceived spousal support, and psychological complications in head and neck cancer: evaluating their association in a longitudinal study*,” was published as an original research article. Head and neck cancer (HNC) patients have generally been examined in response to negative psychological influences such as social isolation, depression, and anxiety; they have rarely been examined for positive outcomes. The study is a well-designed longitudinal study examining possible positive changes (PTG, hope, and optimism). Perceived spousal support and psychological complications (such as depression, anxiety, and post-traumatic stress symptoms) across time. It was observed that there was an increase in post-traumatic growth and perceived spousal support over time. In the time between the first and second data collection, HNC participants reported fewer psychological difficulties (depression, anxiety, and post-traumatic stress symptoms) and higher scores from positive psychology variables (PTG, hope, and optimism) and perceived spousal support. Over time, a higher level of PTG was attributed to greater hope and perceived spousal support. On the other hand, over time, a lower level of PTG was linked to a higher severity of anxiety symptoms. The female gender moderated the association between the severity of anxiety symptoms and PTG; however, this association was not moderated by hope or perceived spousal support. In order to increase the degree of hope and perceived spousal support and lessen the severity of anxiety symptoms, it is crucial to include psychosocial interventions in the treatment regimen, which will, in turn, help HNC patients develop PTG. In addition, the results demonstrated that cultivating positive psychological traits may result in greater resilience and development in the face of adversity.

The second article, authored by Collazo-Castiñeira et al. and titled “*Prediction of post-traumatic growth in the face of the COVID-19 crisis based on resilience, post-traumatic stress, and social participation: a longitudinal study*,” was published as an original research article. The study aims to investigate PTG, resilience, and post-traumatic stress symptoms (PTSS) over three different periods (March, July, and November 2020). There were no significant temporal shifts in the roughly 20% of the sample with moderate-to-high PTG levels. The predictive model demonstrated that PTSS mediated the inverse relationship between resilience and PTG, and this model explained 19% of the variance in PTG. Moreover, engagement in extracurricular activities predicted PTG. Higher PTG was seen in women, younger people, people who had lost their jobs, and those who had experienced COVID-19 symptoms or the death of a loved one. It can be said that people have felt better, but that has not stopped them from experiencing negative symptoms. However, after 8 months, participants showed signs of PTG, suggesting that this shift was not fleeting. Since the COVID-19 crisis is ongoing and the situations at play are dynamic, a long-term analysis of the relationship between PTG and the experiences contributing to its development (e.g., loss of a loved one and participation in social activities) was examined. In this regard, the results revealed that the dynamic nature of traumatic events might contribute to PTG. Findings also highlight the significance of positive experiences like social activities in fostering PTG in the wake of trauma. Finally, how PTG and its underlying mechanisms can be predicted is one of the field's most important and debated issues. The study was aimed at explaining issues.

The third article, authored by Titlestad et al. and titled “*Paths to positive growth in parents bereaved by drug-related death: a mixed-method study*,” was published as an original research article. Drug-related death (DRD) is one of the stressful events, and psychological effects on parents are less likely to be examined. Considering constructive shifts among parents after other tragic events like a child's death from sudden infant death syndrome (SIDS), parents' post-traumatic growth after a DRD was studied using a mixed-method approach to grasp its complexity better. Both a survey and in-depth interviews were conducted in the present study. In the quantitative study, the impact of everyday functioning (WSAS), self-efficacy (GSE-SF), social support (CSS), and extended mourning symptoms (PG-13) on post-traumatic growth short form (PTGI-SF) was investigated using hierarchical multiple regression. Self-efficacy and social support, and growth after traumatic experiences were associated. Also, the “New possibilities” subscale of PTGI-SF had the lowest mean score; many interviewed parents placed a premium on exploring other career choices. Also, item analysis of the PTGI-SF was conducted, that the item “I discovered that I'm stronger than I thought I was” ranked highest on the post-traumatic growth scale. On the other hand, the item “I am able to do better things with my life” ranked lowest. Thoughtful thematic analysis of the qualitative data revealed two overarching themes: (I) novel outlooks on life and (II) novel ways forward. When the findings from the survey with those from the interviews were integrated, a statistically significant correlation was found between surveys and the interviews yielded. Even before their kid died, parents who had dealt with the hardship of caring for an addict noted how their lives had improved. On an individual level, the aftereffects of exposure to negative stereotypes, feelings of inadequacy, and obsessive, repetitive thinking may all work to stunt the healing process. It is noted that increased social support facilitates positive outcomes for growing from adversity. The study demonstrates that helping parents who have gone through a DRD build their social networks and sense of efficacy is considered to be important.

The fourth article, authored by Koutná et al. and titled “*Posttraumatic stress and growth in adolescent childhood cancer survivors: links to quality of life*,” was published as an original research article. The manuscript focuses on the widely discussed and clinically relevant question of the association of post-traumatic stress (PTSS) and post-traumatic growth (PTG) with overall psychosocial adjustment (Quality of life QOL) in long-term childhood cancer survivors. Using a cohort of 172 survivors, it analyzes the associations of stress and growth with various dimensions of quality of life in two age groups (children and adolescents) suffering from pediatric cancer. After controlling age, gender, and time off treatment, the relationship of PTSS and PTG with QOL was significant by regression analyses in a sample of adolescent cancer survivors. However, negative relationships between PTSS and QOL were found, but the relationships between QOL and PTG were insignificant in children. The results illustrate that the relationship between post-traumatic growth and overall adaptation may take different forms at different developmental stages and offer a new perspective on the possible implications of the relationships found.

The fifth article, authored by Blom et al. and titled “*Sub-groups (profiles) of individuals experiencing post-traumatic growth during the COVID-19 pandemic*,” examined PTG in the context of the negative consequences of the COVID-19 pandemic. The main aim was to assess whether distinct sub-groups of individuals experiencing PTG could be identified by how they appraised and coped with the pandemic. They selected a sample of 392 individuals from the general population who had experienced moderate degrees of pandemic-related PTG. Authors have identified two sub-groups that appraised and coped with the pandemic differently. The first resilient group was characterized by increased coping flexibility and greater use of positive reappraisal. Higher levels of stressfulness and greater use of rumination characterized the second stressed group.

The sixth article, authored by Chen et al. and titled “*Perceived posttraumatic growth after interpersonal trauma and subsequent well-being among young Colombian adults: a longitudinal analysis*,” was published as a brief research report. The relationships between perceived PTG and wellbeing across time, especially across domains of functioning, have been the subject of few rigorous investigations. The study is a three-wave longitudinal study of 636 Colombian young adults to investigate the links between perceived PTG and seventeen outcomes measuring mental, social, and physical health. These outcomes included self-rated mental health, meaning in life, happiness, and satisfaction with one's own life. Perceived PTG assessed in Wave 2 was strongly associated with enhancements in one or more aspects of each wellbeing domain assessed in Wave 3 about 6 months later. This was found using an outcome-wide analytic design that included steps to control for potential confounding and reverse causation by adjusting for a range of covariates assessed in Wave 1. The results of this study provide longitudinal data that suggests an association between perceived PTG and improved wellbeing.

The seventh article, authored by Menger et al. and titled “*The nature and content of rumination for head and neck cancer survivors*,” was published as an original research article. Head and Neck Cancer (HNC) can be scary to diagnose and treat. Constant difficulties with everyday activities like speaking and eating may persist after cancer therapy. Compared to survivors of other malignancies, those with an HNC often do worse emotionally. Post-traumatic growth (PTG) is associated with improved health-related quality of life, and it occurs in some people who have survived HNC. Few studies have examined the path toward PTG in cancer but doing so might help shape therapies for better health outcomes in HNC and the avoidance of post-traumatic stress disorder (PTSD). There is general agreement that it helps to be able to think about and process past trauma. Twenty cancer survivors from a variety of HNC subtypes participated in this qualitative research to better understand the impact of this disease and its treatment on rumination. The results provide light on the kind and subject matter of rumination that occurs when HNCs reflect on their experiences. Their findings imply that many thoughts that arise after HNC are fleeting. The anxious and invasive thoughts plaguing us as children often give way to more introspective ruminating as we age. Some HNC survivors are at a higher risk of PTSS because they dwell excessively on the most unpleasant or stressful parts of their treatment.

The eighth article, authored by Yasdiman et al. and titled “*Examining the protective influence of post-traumatic growth on interpersonal suicide risk factors in a 6-week longitudinal study*,” was published as a brief research report. Although suicide is avoidable, it nonetheless claims the lives of about 703,000 individuals worldwide yearly, according to the World Health Organization. Accordingly, to develop evidence-based suicide prevention measures, it is essential to look at risk and protective variables. Post-traumatic growth (PTG) has recently gained attention as a protective factor against suicide. PTG is described as beneficial psychological changes from coping with stressful situations. For instance, prior studies linked PTG with reduced suicide ideation, showing that the ability to draw positive lessons from adversity (i.e., perceive PTG) might serve as a protective factor against the development of suicidal musings. However, the research does not provide a clear explanation for the mechanism behind this association. Perceived burdensomeness (i.e., feeling like a burden to others) and thwarted belongingness (i.e., a sense of failed connectivity) are two interpersonal suicide risk variables that have recently been linked negatively to PTG in cross-sectional studies. In the present publication, we followed up on that study and looked into the connection between the two variables using a longitudinal methodology. The results demonstrated that PTG mediated the reductions in burdensomeness from recent negative experiences 6 weeks later. Our research has implications for suicide prevention and may be used in clinical settings to lessen the effect of negative judgments of burdensomeness and thereby minimize suicidal thoughts.

The ninth article, authored by Moore et al. and titled “*Growth and hope after loss: how TAPS facilitates post-traumatic growth in those grieving military deaths*,” was published as an original research article. Suicide bereavement research has mostly focused on psychopathology. Investigating the potential for one's own development in the midst of an upsetting and traumatic experience is the focus of a new subfield within the field of positive psychology, which provides an additional avenue for better comprehending the implications of the event in question. This study also suggests that assisting others promotes personal development and recovery. When asked about one's mental health, peer mentors of the recently bereaved reported higher levels of PTG. Participation in Tragedy Assistance Program for Survivors (TAPS) and resilience were shown to be reliable and favorable predictors of all subtypes of PTG.

The tenth article, authored by Bakaitytè et al., examined the path from the centrality of an event to PTG, involving intrusive and deliberate rumination and self-blame, as a coping strategy in women survivors of intimate partner violence (IPV). In order to reach this aim, a sample of two hundred women with a history of IPV were recruited, and a series of self-report measures were administered. The path analysis results showed that the event's higher centrality was related to higher levels of intrusive rumination, which was positively related to self-blame and deliberate rumination, eventually leading to PTG. Indirect effects from the centrality of an event to PTG through intrusive and deliberate rumination and from intrusive to deliberate rumination through self-blame were also detected.

The last article, authored by Dominick and titled “*Changes in post-traumatic growth, core belief disruption, and social support over the first year of the COVID-19 pandemic*,” was published as an original research article. This manuscript explores the relative impact of various sources of social support, including support from pets and various coping strategies on post-traumatic growth (PTG) over the first year of the COVID pandemic and a highly contentious political election. Data were collected at a four-time interval from April 2020 (T1) until April 2021 (T4). Although there was no statistically significant change in overall PTG between Time 1 and Time 4, there was a substantial rise in core belief disruption, a reduction in the use of coping techniques, and an increase in the feeling of personal strength and new possibilities. Pet owners, those who knew someone hospitalized with COVID-19, and people who knew someone who died from COVID-19 all had higher PTG. People who saw the incident as concluded had greater PTG than those who saw it as continuing, even though people's ratings of COVID-19 or politics as the most stressful event at Time 4 did not correlate with variations in PTG. Personal exposure to or immunization against COVID-19 did not correlate with different rates of post-traumatic growth. Core belief disruption and social support, as well as support from video conferencing, predicted PTG at Time 4 for both the complete sample and the pet owners alone group. Disrupting fundamental beliefs, focusing on problems, and avoiding triggers all predicted PTG at time four. The PTG theoretical model serves as the basis for discussing the results. Implications for therapies to promote psychological development are also discussed; they include both standard and non-traditional social support mechanisms, such as distant communication and perceived support from pets. Given the time constraints, learning about the benefits of alternative means of obtaining social support is important for informing intervention techniques targeted at enhancing mental health. The findings of this study provide validity to the PTG theoretical model and shed light on who is more likely to undergo psychological development and which variables impact this process.

The last article by Chin et al. aimed to propose a theoretical framework integrating three areas of research: race-based trauma, PTG, and racial identity narratives. Based on the work on Black and Asian American identity and integrating theory and research on historical trauma and PTG, the framework presented by the Authors posits that the transformation of externally imposed narratives into more authentic, internally generated ones can serve as an important influence that sparks PTG after racial trauma. Strategies and tools that enact the cognitive processes of PTG, including writing and storytelling, are finally suggested by the Authors as ways to promote PTG in response to racial trauma.

In conclusion, post-traumatic growth has been investigated with a variety of traumatic events. The results indicate that people may recover and even thrive despite experiencing traumatic events, including head and neck cancer, childhood cancer, suicide, drug overdose mortality, the COVID-19 Crisis, interpersonal trauma, relationship abuse, and racial trauma. Several papers have discussed the numerous personal and societal aspects that contribute to PTG and the ways in which PTG and its associated domains evolve through time. As a result, healthcare professionals and researchers must prioritize the prevention, assessment, and treatment of mental health concerns among people dealing with several traumatic incidents.

## Author contributions

All authors listed have made a substantial, direct, and intellectual contribution to the work and approved it for publication.
